# Electrical Modeling and Characterization of Graphene-Based On-Chip Spiral Inductors

**DOI:** 10.3390/mi13111829

**Published:** 2022-10-26

**Authors:** Da-Wei Wang, Meng-Jiao Yuan, Jia-Yun Dai, Wen-Sheng Zhao

**Affiliations:** 1 Zhejiang Provincial Key Lab of Large-Scale Integrated Circuit Design, School of Electronics and Information, Hangzhou Dianzi University, Hangzhou 310018, China; 2 Science and Technology on Monolithic Integrated Circuits and Modules Laboratory, Nanjing 210016, China

**Keywords:** graphene, on-chip spiral inductor, circuit model, kinetic inductance, quantum resistance

## Abstract

This paper investigates the electrical performance of graphene-based on-chip spiral inductors by virtue of a physics-based equivalent circuit model. The skin and proximity effects, as well as the substrate loss effect, are considered and treated appropriately. The graphene resistance and inductance are combined into the circuit model. It is demonstrated that the electrical characteristics of the on-chip square spiral inductor can be improved by replacing copper with graphene. Moreover, graphene exhibits more effectiveness in improving the inductance in tapered inductors than uniform ones.

## 1. Introduction

With the improvement of CMOS technologies, radio-frequency integrated circuits (RF ICs) have become possible and have drawn increasing attention in the past decades. To make RF ICs lightweight, multi-functional, and low-cost, on-chip spiral inductors with high inductance density and low power consumption have been widely utilized in the design of low-noise amplifiers, voltage-controlled oscillators, and filters [1]. 

In order to improve the inductor performance, it is intuitive to pursue high inductance density and quality factors. There are various factors affecting inductor characteristics, such as substrate resistivity and metal thickness. Increasing metal thickness can improve the quality factor by minimizing Ohmic losses but is counter-productive at high frequencies due to accentuated proximity effect loss [2]. The magnetic field from all turns of a spiral inductor accumulates in the middle area, thereby resulting in severe current crowding in the inner turns. As the current crowding can be mitigated with narrow and widely spaced inner turns, a tapered spiral inductor was proposed in [3] to improve the quality factor without consuming extra area.

To facilitate the miniaturization of RF ICs, the scaling down of on-chip spiral inductors is inevitable [4]. However, there exists an inherent limitation in the scalability of conical spiral inductors, as the inductance value is limited by the laws of electromagnetic induction [5]. This is, the magnetic flux is proportional to the surface area, and the magnetic inductance cannot be scaled to retain desired inductance density. 

Carbon nanomaterials, including carbon nanotube (CNT) and graphene, have been proposed as promising alternative candidates for interconnected applications. They have been demonstrated to have unique physical properties such as a long mean free path (MFP), extremely high ampacity, and large thermal conductivity, and they exhibit superior performance and reliability to traditional metal wires [6,7,8,9]. More importantly, carbon nanomaterials possess large kinetic inductance, which makes them suitable for building inductors in future scaled RF ICs. It was proven that CNT-based on-chip spiral inductors can provide better quality factors than their Cu counterparts [10]. In comparison with CNT, graphene is more compatible with the traditional CMOS process [11], and there are ways to control graphene chirality and doping levels [12]. Graphene-based on-chip spiral inductors were explored in-depth recently [5,13]. It was experimentally demonstrated that the graphene kinetic inductance is beneficial for improving the inductor performance. Although the graphene-based inductors were investigated numerically in [13], the anomalous skin effect could be neglected as it mainly affects the electrical characteristics beyond 100 GHz, and the modeling methodology could be therefore simplified. 

This paper aims to provide a simple circuit model for investigating graphene-based inductors. Firstly, the equivalent circuit model of an on-chip tapered spiral inductor is developed, with the proximity effect and substrate loss taken into account. The quantum contact resistance, scattering resistance, and kinetic inductance of graphene ribbon (GR) are combined into the model to explore the electrical characteristics. By virtue of the circuit model, the electrical performance of graphene-based inductors is investigated, with some guidance provided for future development. The rest of this paper is organized as follows. Section 2 presents the geometry of the on-chip tapered spiral inductor and its physics-based equivalent circuit model. The graphene kinetic inductance is discussed in Section 3, and it is combined into the model to explore the inductor characteristics. The performance analysis of graphene-based spiral inductors is carried out in Section 4. Some conclusions are finally drawn in Section 5.

## 2. Circuit Model of On-Chip Square Spiral Inductor

**Figure 1:**
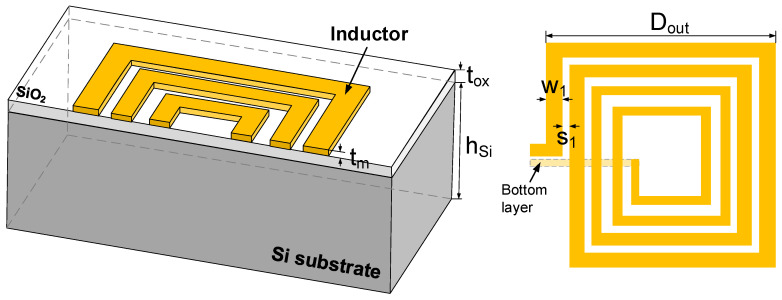
Schematic of on-chip square spiral inductor.

On-chip spiral inductors can be divided into rectangular, square, circular, hexagon, and octagonal types according to their geometries. Due to their simple configuration, square spiral inductors are widely used in the design of RF ICs, as shown in Figure 1. In the figure, $D_{\mathrm{out}}$ is the outer diameter, and $w_{1}$ and $s_{1}$ denote the width and spacing of the outermost turn, respectively. For the tapered spiral inductor, the difference in the width between adjacent turns is defined as taper, with the pitch kept constant. Accordingly, the ith turn has a width of $w_{i}=w_{1}-\left(i-1\right)\cdot taper$, and its spacing with an adjacent inner turn is $s_{i}=s_{1}+\left(i-1\right)\cdot taper$.

**Figure 2:**
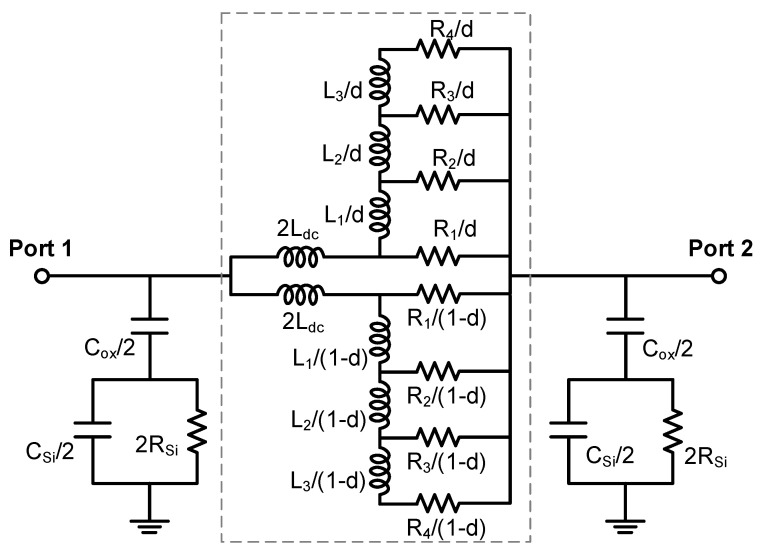
Equivalent circuit model.

Figure 2 shows the π-equivalent circuit model of the on-chip tapered spiral inductor, which is composed of oxide capacitance $C_{\mathrm{ox}}$, substrate capacitance $C_{\mathrm{Si}}$, and substrate resistance $R_{\mathrm{Si}}$ [14]. To obtain the inductance value accurately, the widely applied Greenhouse formulas of a planar spiral inductor are adopted, and the total inductance of the spiral inductor is calculated by

(1) $$L_{\mathrm{dc}}=L_{\mathrm{self}}+\sum^{\;}M_{+}+\sum^{\;}M_{-}$$

where $L_{\mathrm{self}}$ is the self-inductance of a metal line, and $M_{+}$ and $M_{-}$ are mutual inductances between two lines.

The dc resistance of a spiral inductor is calculated by

(2) $$R_{\mathrm{dc}}=\frac{1}{\sigma_{m}t_{m}}\overset{N}{\underset{i=1}{\sum }}\frac{l_{i}}{w_{i}}$$

where $\sigma_{m}$ is the metal conductivity. To accurately evaluate the inductor characteristics, the frequency-dependent effect should be treated appropriately. A ladder network marked by the dashed box in Figure 2 is added for modeling skin and proximity effects [15]. At low frequencies, the current is uniformly distributed inside the inductor, while the current accumulation occurs at the surface layer of the inductor at high frequencies. The high-frequency resistance $R_{1}$ is calculated by

(3) $$R_{1}=\frac{1}{2\sigma_{m}t_{m}\delta_{\max}}\overset{N}{\underset{i=1}{\sum }}l_{i}$$

where $\delta_{\max}$ denotes the skin depth at the maximum operating frequency $f_{\max}$, and it is given as $\delta_{\max}=1/\sqrt{\pi \mu \sigma_{m}f_{\max}}$. The calculations of other resistances and inductances can refer to [15]. Further, 2-branch networks are employed to improve the model accuracy, with the proximity factor defined as [15]

(4) $$d=L_{\mathrm{dc}}\cdot {\left[2L_{\mathrm{dc}}-\frac{1}{2I}\overset{N}{\underset{i=1}{\sum }}w_{i}l_{i}\left(B_{i,i}+B_{i,\mathrm{others}}\right)\right]}^{-1}$$

where I is the excitation current, and $B_{i,i}$ and $B_{i,\;\mathrm{others}}$ represent the magnetic fields due to the ith turn and the other turns except ith turn, respectively. The minimum and maximum values of d are 0.5 and 1, which correspond to the cases of no proximity effect and maximum proximity effect [16]. 

The oxide and silicon capacitance are calculated by

(5) $$C_{\mathrm{ox}}=\frac{\varepsilon_{\mathrm{ox}}}{t_{\mathrm{ox}}}\overset{N}{\underset{i=1}{\sum }}w_{i}l_{i}$$

(6) $$C_{\mathrm{Si}}=2\varepsilon_{\mathrm{Si}}\overset{N}{\underset{i=1}{\sum }}\frac{w_{i}l_{i}}{2h_{\mathrm{Si}}+\sqrt{\frac{w_{i}l_{i}}{\pi }}-\sqrt{4h_{\mathrm{Si}}^{2}+\frac{w_{i}l_{i}}{\pi }}}$$

(7) $$R_{\mathrm{Si}}=\varepsilon_{\mathrm{Si}}/\left(\sigma_{\mathrm{Si}}C_{\mathrm{Si}}\right)$$

where $\varepsilon_{\mathrm{ox}}$ is the permittivity of the oxide layer, and $\varepsilon_{\mathrm{Si}}$ and $\sigma_{\mathrm{Si}}$ are the permittivity and conductivity of the silicon substrate, respectively. 

**Figure 3:**
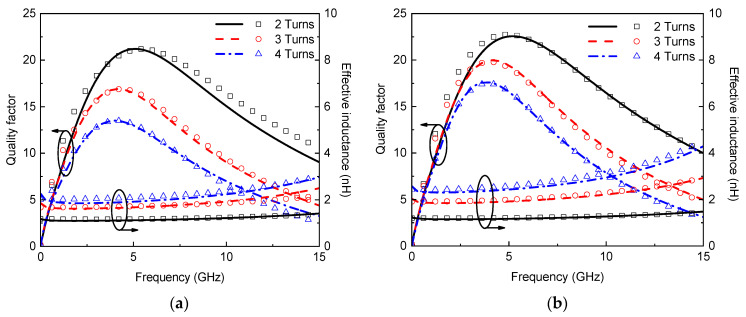
Effective inductance and quality factor of on-chip (**a**) uniform and (**b**) tapered spiral inductors (lines: simulation; symbols: model).

To evaluate the circuit model, a set of square spiral inductors are simulated using a full-wave electromagnetic simulator, i.e., ANSYS HFSS (2021 version, Ansys, Inc., Canonsburg, PA, USA). In the simulation; the silicon conductivity is 10 S/m, and the geometrical parameters are as follows: $D_{\mathrm{out}}=200\;\mu m$; $w_{1}=13\;\mu m$; and $s_{1}=7\;\mu m$. For the tapered spiral inductor, taper=2 μm/turn. The effective inductance and quality factor are obtained from the simulated *Y*-parameters and plotted in Figure 3:

(8) Q=Im(Y11)Re(Y11)

(9) Leff=12πfIm(1Y11)

It is evident that the results obtained by the circuit model agree well with the simulated results. The uniform spiral inductor possesses smaller dc resistance than the tapered one but has larger high-frequency resistance due to skin and proximity effects. The effective inductance of the spiral inductor can be increased by introducing tapering, and therefore, the quality factor can be improved. 

## 3. Modeling of Graphene-Based Inductors

According to Faraday’s law of electromagnetic induction, the current change in the metal turns of a spiral inductor produces time-varying magnetic flux. As the magnetic flux is proportional to the inductor area, the decreased size degrades the inductance density, thereby limiting the scaling of RF ICs. Kinetic inductance, which originates in the kinetic energy required by mobile charge carriers in alternative electromotive force, is usually ignored in conventional metals due to their small relaxation time and large conducting channel number [5]. However, the momentum relaxation time of graphene is on the order of picoseconds, and therefore, graphene possesses large kinetic inductance, which makes it suitable for building on-chip spiral inductors in future scaled RF ICs.

### 3.1. GR Impedance

**Figure 4:**
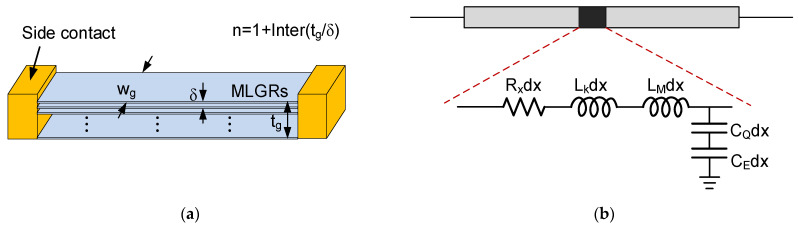
(**a**) Schematic of multilayer GR interconnect and its (**b**) equivalent distributed circuit model.

Figure 4a shows the structure of a multilayer GR interconnect with side contacts [12]. In the figure, $w_{g}$ and $t_{g}$ are the width and thickness, δ is the spacing between adjacent graphene layers, and the number of graphene layers is calculated as $n=1+\mathrm{Inter}\left(t_{g}/\delta \right)$, where “Inter(·)” denotes that only the integer part is considered [10]. The corresponding equivalent circuit model of a multilayer GR is depicted in Figure 4b. The number of conducting channels per layer of graphene sheet is given as [7]

(10) $$N_{\mathrm{ch}}=\overset{n_{C}}{\underset{i=0}{\sum }}{\left(1+e^{\frac{E_{i}-E_{F}}{k_{B}T}}\right)}^{-1}+\overset{n_{V}}{\underset{i=0}{\sum }}{\left(1+e^{\frac{E_{i}-E_{F}}{k_{B}T}}\right)}^{-1}$$

where the first and second summations on the right-hand side of (10) represent the contributions of the conduction subbands and valence subbands, respectively, $k_{B}$ is the Boltzmann constant, T is the temperature, $E_{F}$ is the Fermi energy, and $E_{i}$ denotes the ith conduction (valence) subbands with the lowest (highest) energy. For GR with $w_{g}>10\;\mathrm{nm}$ and $E_{F}>0.1\;\mathrm{eV}$, $N_{\mathrm{ch}}$ has a linear relationship with $w_{g}$ and $E_{F}$, i.e., $N_{\mathrm{ch}}=\alpha w_{g}E_{F}$, where $\alpha =1.2\;{\mathrm{eV}}^{-1}\cdot {\mathrm{nm}}^{-1}$ is the fitting coefficient [17]. 

The kinetic inductance of a single-layer GR is given by [17]

(11) $$L_{K}\approx \frac{8\;\mathrm{nH}/\mu m}{N_{\mathrm{ch}}}=\frac{8\;\mathrm{nH}/\mu m}{\alpha w_{g}E_{F}}$$

**Figure 5:**
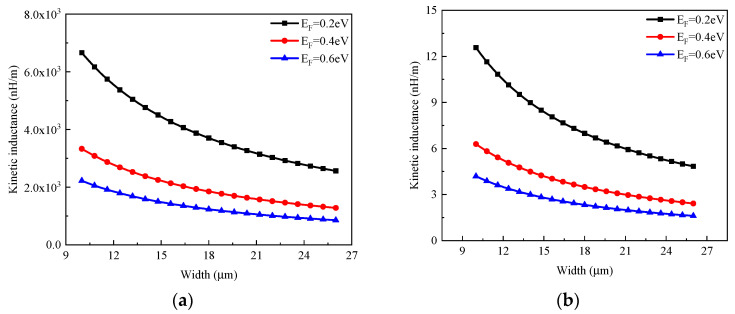
Kinetic inductances of (**a**) single-layer and (**b**) multilayer GRs.

It is worth noting that graphene tends to graphite as the layer number increases [18]. However, decoupled graphene layers were experimentally demonstrated in [19], and it is expected that a multilayer GR with a certain thickness can be realized in the future. Therefore, the multilayer GR is regarded as a stack of single-layer GRs in this study, and its inductance is calculated as $L_{K}\approx 8\;\mathrm{nH}/\mu m/\left(n\alpha w_{g}E_{F}\right)$. Figure 5 shows the kinetic inductances of single- and multilayer GRs. It is evident that the GR inductance decreases with $E_{F}$, and single-layer GR exhibits much larger kinetic inductance than multilayer GR. However, the single-layer GR is not suitable for building inductors due to its ultrahigh resistive loss.

**Figure 6:**
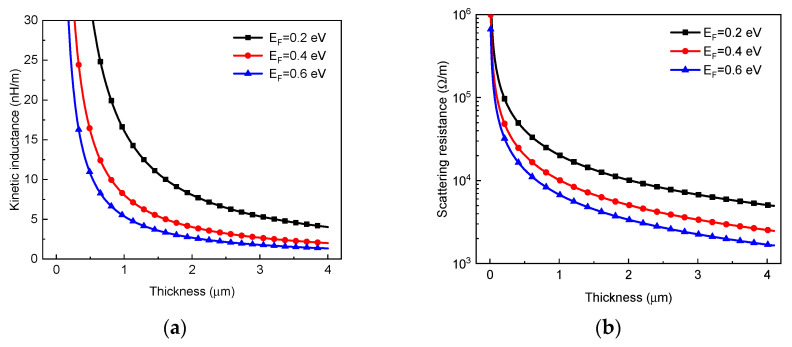
(**a**) Kinetic inductances and (**b**) resistance of GR interconnect versus thickness for different Fermi levels.

As shown in Figure 6a, the kinetic inductance of multilayer GR is in inverse proportion to the layer number and thereby decreases with increasing thickness. For building an on-chip spiral inductor, the GR length is usually larger than the effective MFP $\lambda_{\mathrm{eff}}$, and therefore, the scattering resistance of GR can be approximated as [17]

(12) $$R_{S}\approx \frac{12.9\;k\Omega }{nN_{\mathrm{ch}}\lambda_{\mathrm{eff}}}=\frac{12.9\;k\Omega }{n\alpha w_{g}E_{F}\lambda_{\mathrm{eff}}}$$

where $\lambda_{\mathrm{eff}}$ is related to various scattering mechanisms, and it has a dominating effect in determining the signal transmission performance [7]. As shown in Figure 6b, the GR resistance decreases with the thickness and Fermi level. To improve the graphene conduction, it is necessary to increase the Fermi level and MFP by appropriate doping techniques [20]. It is worth noting that the fabricated GRs usually cannot come up to theoretical predictions due to manufacturing errors and defects.

### 3.2. Spiral Inductors

Although an anomalous skin effect exists in GR due to the large ratio of in-plane to out-plane MFP, it mainly appears as the operating frequency exceeds several tens of gigahertz [13]. Therefore, the anomalous skin effect can be neglected, as it has little influence on the inductor characteristics in the frequency range of interests of this study. Considering the kinetic inductance, the dc inductance of the graphene-based spiral inductor is given by

(13) $$L_{\mathrm{dc}}=L_{\mathrm{self}}+\sum^{\;}M_{+}+\sum^{\;}M_{-}+L_{K,t}$$

where $L_{K,t}$ represents the total kinetic inductance, and it can be calculated by

(14) $$L_{K,t}=\left(\frac{1}{n\alpha E_{F}}\overset{N}{\underset{i=1}{\sum }}\frac{l_{i}}{w_{\mathrm{eff},i}}\right)\times 8\mathrm{nH}/\mu m$$

The effective width $w_{\mathrm{eff},\;i}$ is equal to two times the skin depth [5]. The effective conductivity of multilayer GR is given by

(15) $$\sigma_{\mathrm{eff}}=\frac{1}{R_{S}wt}=\frac{n\alpha E_{F}\lambda_{\mathrm{eff}}}{t}\times \frac{1}{12.9\;k\Omega }$$

**Figure 7:**
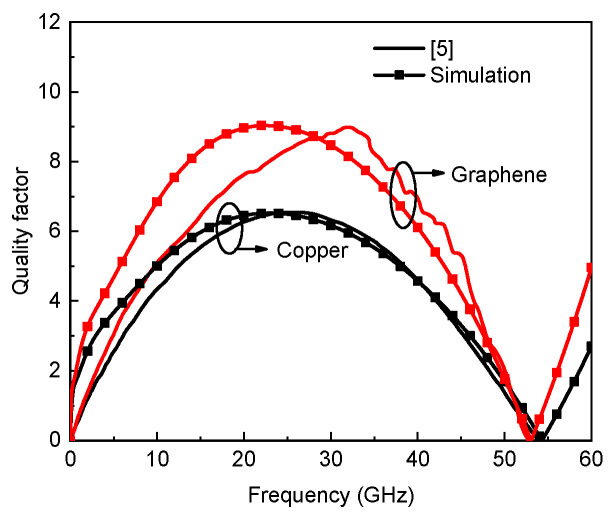
Quality factors of Cu- and graphene-based inductors.

Note that the width in both the numerator and denominator cancel out, and the effective conductivity would be irrelevant to the width. Therefore, the conductivity can be treated as a constant for a specific GR line in the modeling of graphene-based inductors. By utilizing the above equations and circuit model shown in Figure 2, the electrical characteristics of graphene-based on-chip spiral inductors can be investigated. The quantum contact resistances are incorporated into the ends of the model, but it has little influence on the inductor performance. The metallic vias in graphene-based inductors could be made of Co [12]. Note that the modeling methodology can also be applied to other spiral inductor structures, such as circular, hexagon, and octagonal types. To verify the model, the quality factor of the graphene-based spiral inductor is obtained and compared with the experimental results in [5]. The geometrical parameters are as follows: $D_{out}=200\;\mu m$, w=25 μm, and s=5 μm. It can be seen from Figure 7 that the modeling results basically agree with the experimental results, and the deviation may be attributed to incorrect geometrical parameters, which will be investigated in the next study. 

## 4. Results and Discussion

**Figure 8:**
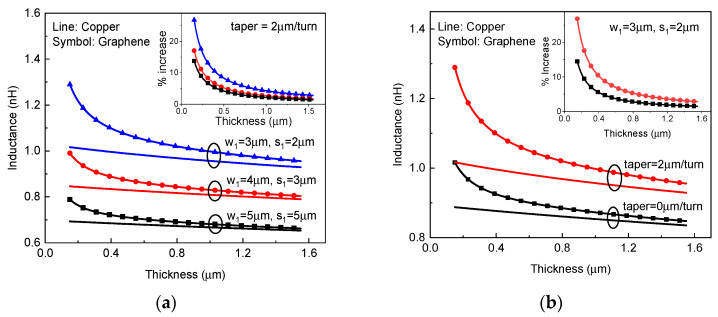
Inductance versus thickness for copper and graphene-based on-chip spiral inductors with different (**a**) width, spacing, and (**b**) taper. The inset plots the percentage increase in inductance of graphene inductor to copper inductor.

For graphene-based on-chip spiral inductors, the inductance mainly comes from the magnetic inductance and the kinetic inductance, which originates in the kinetic energy required by mobile electrons, and its value depends on the number of conduction channels. In order to characterize the influence of kinetic inductance, the inductances of copper and graphene-based on-chip spiral inductors made of copper and graphene are plotted in Figure 8. It is evident that graphene becomes superior to copper for building on-chip inductors with a decreasing geometric size (e.g., thickness and width), implying that graphene is more suitable for the applications of future scaled RF ICs. Moreover, as shown in Figure 8b, the advantage of a graphene-based inductor over its copper counterpart can be strengthened by increasing taper. This is mainly because the inner ring of a graphene-based tapered spiral inductor could provide larger kinetic inductance due to its smaller width than that of a uniform inductor. 

In general, graphene doping can increase the Fermi level and thereby decrease the resistive loss. Moreover, the coupling effect between adjacent graphene layers can be alleviated and finally cancelled by intercalation doping. Therefore, alternate dopants such as AsF_5_, Br_2_, FeCl_3_, and KI are continually being investigated [21,22]. Among these dopants, Br is easy to diffuse into graphene layers, and its doping process is relatively simple and efficient. The average thickness increment of Br-doped multilayer graphene is about 6.7% of the original thickness [5], i.e., the average layer spacing between adjacent graphene layers is about 0.3628 nm. As doping is a process of charge transfer, the carrier density and conductivity vary with the doping time. As the doping time exceeds 70 min, the resistivity of Br_2_-doped graphene becomes lower than that of bulk copper, and the Fermi level reaches 0.5 eV [23]. Here, two cases of Br_2_-doped graphene are considered, i.e., the conductivities are set as 5 × 10^7^ S/m and 10^8^ S/m, respectively. 

**Figure 9:**
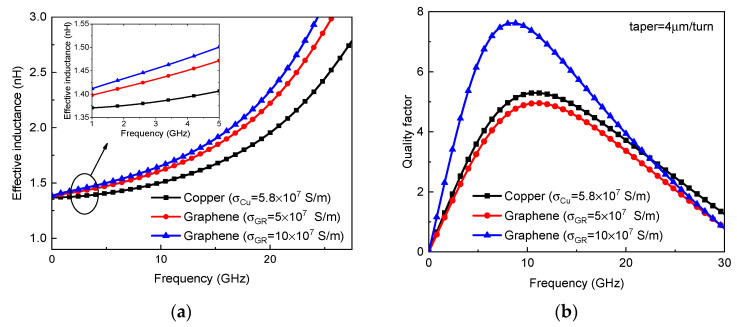
(**a**) Effective inductance and (**b**) quality factors of on-chip tapered spiral inductors made of copper and graphene.

**Figure 10:**
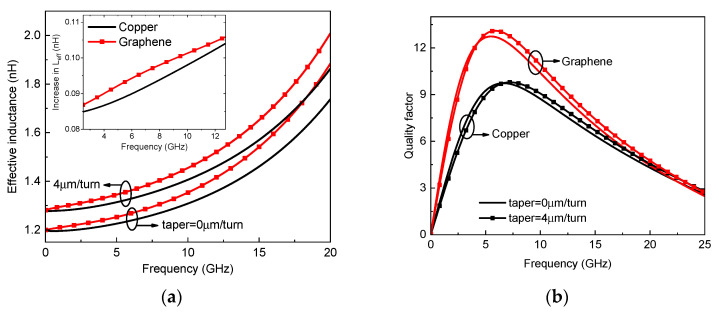
(**a**) Effective inductance and (**b**) quality factor of on-chip uniform and tapered spiral inductors.

**Figure 11:**
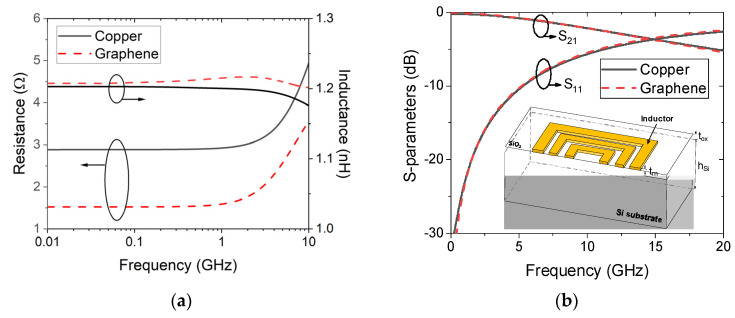
(**a**) Impedances (the model in the dashed box of Figure 2 and (**b**) scattering parameters of on-chip uniform spiral inductors.

By virtue of the circuit model in Figure 2, the effective inductance and quality factor of on-chip tapered spiral inductors made of copper and Br_2_-doped graphene are plotted in Figure 9. It is evident that the effective inductance can be improved by replacing copper with graphene. For graphene with a conductivity of 5 × 10^7^ S/m, the quality factor of a graphene-based spiral inductor is slightly lower than that of its copper counterpart. However, as the graphene conductivity exceeds 10^8^ S/m, the quality factor can be significantly improved due to the reduction of metal resistive loss. Figure 10 shows the effective inductance and quality factor of an on-chip spiral inductor with different values of taper. It can be seen from Figure 10a that the effective inductance can be increased by increasing taper. However, as the inductor characteristics are affected by various factors, such as substrate loss, the quality factor of a tapered spiral inductor is slightly larger than that of a uniform one. Further, the frequency-dependent impedances and scattering parameters of copper and graphene-based on-chip uniform spiral inductors are plotted in Figure 11. It is evident that GR could provide smaller resistance and a slightly larger inductance than its copper counterpart, but their scattering parameters are comparable due to other influences, such as the substrate loss effect. 

**Figure 12:**
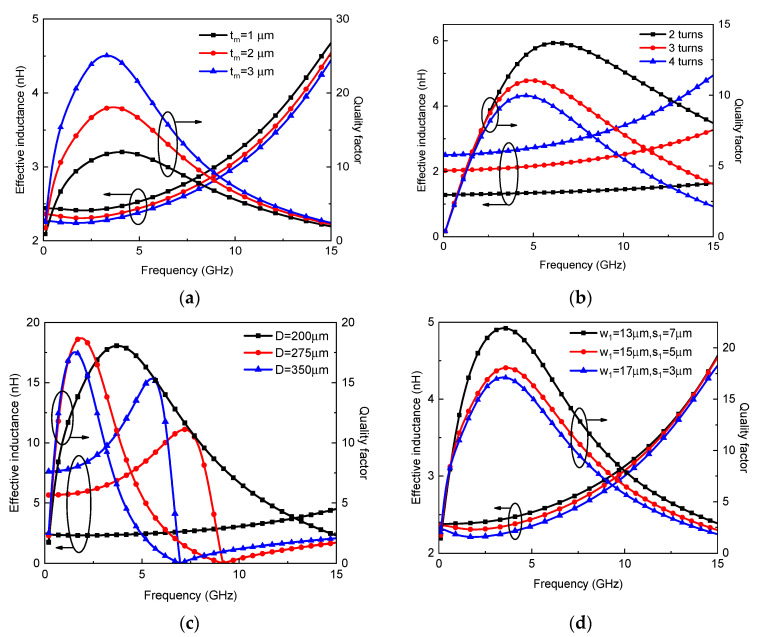
Effective inductance and quality factor of graphene-based on-chip tapered spiral inductors with different (**a**) thicknesses, (**b**) turn numbers, (**c**) outermost diameter, (**d**) width and spacing.

**Figure 13:**
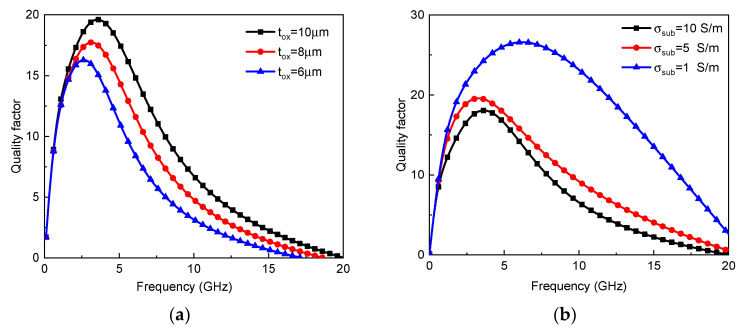
Quality factor of graphene-based on-chip tapered spiral inductors with different (**a**) oxide layer thicknesses and (**b**) substrate conductivities.

Further, the parametric study of graphene-based on-chip spiral tapered inductors was conducted. The influence of thickness on the inductor characteristics is shown in Figure 12a. With the increasing thickness, the effective inductance decreases, while the quality factor increases significantly due to the reduction of metal resistive loss. As shown in Figure 12b, the increase in turn number increases the effective inductance and decreases the quality factor. Similarly, as shown in Figure 12c, the increasing outermost diameter increases the effective inductance. However, the magnetic field penetrating the inductor coil generates current in the substrate, thereby leading to an increase in eddy current loss [24]. The larger the outermost diameter, the higher the eddy current loss. Thus, due to the increase in both effective inductance and eddy current loss, the maximum quality factor is slightly changed with the outermost diameter. Moreover, the self-resonant frequency decreases dramatically with the increasing outermost diameter. Figure 12d shows the characteristics of graphene-based inductors with different widths and spacings. With a decreasing width, the magnetic inductance increases, and the resistive loss is reduced due to the suppressed proximity effect, thereby increasing the maximum quality factor. The influence of oxide layer thickness on the quality factor of a graphene-based on-chip spiral inductor is explored, as shown in Figure 13a. The thicker the oxide layer is, the greater the quality factor is. The self-resonant frequency increases with the increasing oxide layer thickness. Moreover, as shown in Figure 13b, the quality factor increases with the decreasing substrate conductivity due to the reduced substrate loss. By virtue of the circuit model, some guidance is given for the design and fabrication of graphene-based inductors, which is anticipated to be validated in a future experimental study. It is worth noting that although the simulation could give insight into the device’s operation, real-world implementation is inevitable, as the simulation might be too idealistic. Moreover, much effort should be spent in all fields related to the fabrication of graphene-based inductors, including graphene growth and doping.

In summary, the graphene-based on-chip square spiral inductors are investigated by virtue of the circuit model. Although graphene possesses large kinetic inductance, its advantage over copper becomes significant only when the inductor dimensions are smaller than a certain value. This is, the area of the nanoscale inductor can be reduced by replacing copper with GR. On the other hand, the conductivity can be increased by employing doping techniques, thereby improving the inductor quality factor. However, the simulation is realistic, and much effort should be devoted to the fabrication of high-quality GRs. 

## 5. Conclusions

In this paper, the ultimate electrical performance of graphene-based spiral inductors was investigated theoretically. The equivalent circuit model of a traditional on-chip spiral inductor was developed and verified. The graphene kinetic inductance was combined into the model, and the electrical characteristics of on-chip spiral inductors made of copper and graphene were captured and compared. In the modeling procedure, it was found that the quantum contact resistance has little influence on the inductor performance. Graphene could be superior to its copper counterpart for building inductors when the geometry is very small, indicating that graphene is more suitable for future nanoscale RFICs. Moreover, a tapered spiral inductor could better play the advantage of graphene than a uniform one, as the inner ring has larger kinetic inductance. Although graphene possesses high kinetic inductance, the quality factor of the graphene-based inductor may be limited by its resistive loss, and therefore, the intercalation doping technique should be pursued. By virtue of the circuit model, the influences of geometrical parameters on the performance of graphene-based on-chip spiral inductors were finally explored, with several design guidelines provided. 

## Data Availability

Not applicable.
